# Emerging phenotypes linked to variants in *SAMD9* and MIRAGE syndrome

**DOI:** 10.3389/fendo.2022.953707

**Published:** 2022-08-18

**Authors:** Jenifer P. Suntharalingham, Miho Ishida, Ignacio Del Valle, Susanne E. Stalman, Nita Solanky, Emma Wakeling, Gudrun E. Moore, John C. Achermann, Federica Buonocore

**Affiliations:** ^1^ Genetics and Genomic Medicine Research and Teaching Department, UCL Great Ormond Street Institute of Child Health, University College London, London, United Kingdom; ^2^ Department of Pediatrics, Academic Medical Centre, University of Amsterdam, Amsterdam, Netherlands; ^3^ North East Thames Regional Genetic Service, Great Ormond Street Hospital, London, United Kingdom

**Keywords:** SAMD9, FGR, NGS, adrenal insufficiency, endocrinopathies, myelodysplastic syndrome (MDS), MIRAGE syndrome

## Abstract

**Background:**

Heterozygous *de novo* variants in *SAMD9* cause MIRAGE syndrome, a complex multisystem disorder involving Myelodysplasia, Infection, Restriction of growth, Adrenal hypoplasia, Genital phenotypes, and Enteropathy. The range of additional clinical associations is expanding and includes disrupted placental development, poor post-natal growth and endocrine features. Increasingly, milder phenotypic features such as hypospadias in small for gestational age (SGA) boys and normal adrenal function are reported. Some children present with isolated myelodysplastic syndrome (MDS/monosomy 7) without MIRAGE features.

**Objective:**

We aimed to investigate: 1) the range of reported *SAMD9* variants, clinical features, and possible genotype-phenotype correlations; 2) whether SAMD9 disruption affects placental function and leads to pregnancy loss/recurrent miscarriage (RM); 3) and if pathogenic variants are associated with isolated fetal growth restriction (FGR).

**Methods:**

Published data were analyzed, particularly reviewing position/type of variant, pregnancy, growth data, and associated endocrine features. Genetic analysis of *SAMD9* was performed in products of conception (POC, n=26), RM couples, (couples n=48; individuals n=96), children with FGR (n=44), SGA (n=20), and clinical Silver-Russell Syndrome (SRS, n=8), (total n=194).

**Results:**

To date, *SAMD9* variants are reported in 116 individuals [MDS/monosomy 7, 64 (55.2%); MIRAGE, 52 (44.8%)]. Children with MIRAGE features are increasingly reported without an adrenal phenotype (11/52, 21.2%). Infants without adrenal dysfunction were heavier at birth (median 1515 g versus 1020 g; P < 0.05) and born later (median 34.5 weeks versus 31.0; P < 0.05) compared to those with adrenal insufficiency. In MIRAGE patients, hypospadias is a common feature. Additional endocrinopathies include hypothyroidism, hypo- and hyper-glycemia, short stature and panhypopituitarism. Despite this increasing range of phenotypes, genetic analysis did not reveal any likely pathogenic variants/enrichment of specific variants in *SAMD9* in the pregnancy loss/growth restriction cohorts studied.

**Conclusion:**

MIRAGE syndrome is more phenotypically diverse than originally reported and includes growth restriction and multisystem features, but without adrenal insufficiency. Endocrinopathies might be overlooked or develop gradually, and may be underreported. As clinical features including FGR, severe infections, anemia and lung problems can be non-specific and are often seen in neonatal medicine, SAMD9-associated conditions may be underdiagnosed. Reaching a specific diagnosis of MIRAGE syndrome is critical for personalized management.

## Introduction

*SAMD9* (sterile α motif domain-containing protein 9) is a single exon gene that encodes a 1,589-amino acid protein and is located on the long arm of chromosome 7 (7q21.2) in humans, next to its paralogous gene *SAMD9L* (SAMD9-like) in a head-to-toe orientation (1). The molecular role of SAMD9 is not fully elucidated, however it has been shown to be a cytoplasmic protein involved in viral host defense mechanisms, cell proliferation, endosomal fusion and tumor suppression (2–6).

Deleterious loss-of-function variants in *SAMD9* were originally reported to be associated with normophosphatemic familial tumoral calcinosis in rare cases (OMIM: 610455) (7, 8). However, it is now well established that heterozygous gain-of-function variants in *SAMD9* can be identified in children with a complex multisystem condition named MIRAGE syndrome (myelodysplasia, infection, restriction of growth, adrenal hypoplasia, genital (gonadal) phenotypes, and enteropathy) (OMIM: 617053) (9–11). Although this acronym embraces several common features, the range of additional clinical associations is expanding and includes achalasia/gastroesophageal reflux, recurrent intussusception, renal features (focal segmental glomerulus sclerosis), dysautonomia, autoinflammation, learning difficulties, and hypolacrima/corneal anesthesia, amongst other findings (12–21). Disrupted placental development has also been reported, as well as poor post-natal growth (22).

The missense gain-of-function variants causing MIRAGE syndrome result in decreased cell proliferation and growth restriction in *in vitro* model systems, highlighting the innate role of SAMD9 as a growth repressor. Most *SAMD9* variants occur *de novo*, however *SAMD9* patients can inherit variants from asymptomatic parents either due to germline variants or revertant mosaicism events (20, 23). Indeed, dynamic, somatic revertant mechanisms are now established as frequent genomic events in individuals with pathogenic *SAMD9* variants and are often seen in the hematopoietic system/bone marrow. Here, cells with secondary changes that “remove” the primary growth repressive *SAMD9* variant have a clonal growth advantage. These changes include the progressive development of monosomy 7 or deletion of 7q (containing *SAMD9*); secondary loss-of-function variants in *SAMD9* (nonsense, frameshift, missense); or acquired uniparental disomy of the “wild-type” allele (9, 10). Secondary events usually occur *in cis* to ameliorate the effects of the primary gain-of-function variants in the proband and, at least in the hematopoietic lineage, serve to modify disease phenotype. However, when monosomy 7 occurs there is a risk of myelodysplastic syndrome due to loss of *SAMD9*, *SAMD9L*, *GATA2* and other factors, and an additional risk of leukemia if further somatic changes occur.

To date, more than 50 patients have been reported with severe growth restriction phenotypes due to gain-of-function changes in *SAMD9* (9, 10, 12–22, 24–38). Although endocrine features such as primary adrenal insufficiency and gonadal dysgenesis were originally a core part of the syndrome, it is emerging that these features may be more variable. For example, *SAMD9* pathogenic variants have now been identified in individuals with hypospadias (46,XY DSD) and born small for gestational age (SGA), and increasingly in children without primary adrenal insufficiency (22). Other endocrine systems may be affected, such as the thyroid gland. Thus, the endocrine phenotype of MIRAGE syndrome is likely to be more variable than originally described and may comprise additional features not always reported.

We hypothesize that: 1) different *SAMD9* variants, or variants in different domains of the protein, are associated with milder or diverse endocrine phenotypes, with a focus on adrenal insufficiency; 2) severe gain-of-function of SAMD9 may affect placental function and lead to pregnancy loss and recurrent miscarriage (RM); and that 3) pathogenic variants could be found in children with fetal growth restriction (FGR) as the predominant feature. Determining whether *SAMD9* variants cause isolated growth restriction is important as these children could develop monosomy 7, endocrinopathies or immune dysfunction, and could require personalized management such as bone marrow/stem cell transplantation.

## Materials and methods

### Meta-analysis of SAMD9-associated variants/MIRAGE syndrome

A systematic review was undertaken to identify published reports of individuals with SAMD9-associated conditions in the literature. PubMed was searched using the term “SAMD9”, up to May 2022. For this review, a particular focus was put on position and type of variant, pregnancy and growth data, and reported associated endocrine features. All published *SAMD9* variant-carrying individuals included in our study and their related publication link are available here http://doi.org/10.17605/OSF.IO/WMVXY (39).

### Study cohort and ethics

Samples were provided by the Wellbeing of Women Baby Bio Bank (BBB) (http://www.ucl.ac.uk/babybiobank) supported by University College London, Imperial College London and Wellbeing of Women (40) and the Moore Cohort with ethical approval (BBB Research Ethics Committee references: 09/H0405/30 and 09/H0405/30+5; Moore cohort reference: 2001/6029). Five different cohorts were included (**Table 1**): 1) Products of conception (POC) (n=26), spontaneous loss of pregnancy between 9-11 weeks gestation; 2) Recurrent miscarriage (RM) (n=96; 48 couples), couples who experienced three or more consecutive loss of pregnancies before 20 weeks gestation; 3) FGR (n=44), a fetus with growth restriction of unknown etiology *in utero* (41) and in this cohort having a weight less than 3^rd^ centile (42); 4) Small for gestational age (SGA) (n=20), a baby with birthweight below the 10^th^ percentile for gestational age (42); 5) Clinical Silver-Russell Syndrome (SRS) (n=8), based on Consensus guidelines for diagnosis (43) and all double negative for H19 hypomethylation and maternal UPD7.

**Table 1:** Overview of the cohorts included.

Cohort	Number of Individuals	Description	Sequencing Panel
Product of Conception (POC)	26	DNA from spontaneous loss of pregnancies between 9-11 weeks gestation	HaloPlex targeted NGS
Recurrent Miscarriage (RM)	96	DNA from couples who experienced 3 or more consecutive loss of pregnancies before 20 weeks gestation	HaloPlex targeted NGS
Fetal Growth Restriction (FGR)	44	DNA from newborns with a birth weight less than the 3^rd^ percentile	HaloPlex targeted NGS (n=35) and Nonacus Cell3TM Target NGS panel (n=9)
Small for Gestational Age (SGA)	20	DNA from newborns with a birth weight below the 10^th^ percentile	Agilent SureSelect Human All Exon V5 WES
Silver-Russell Syndrome (SRS)	8	DNA from children with sporadic clinical Silver-Russell Syndrome	Nonacus Cell3TM Target NGS panel

Description of individual cohorts analyzed in our study, showing the total number of cases in each group and the sequencing panels used.

NGS, next-generation sequencing; WES, whole exome sequencing.

### DNA extraction and genetic analysis

DNA was isolated from blood leukocytes and POC as previously described (40, 44, 45). DNA samples were subjected to genetic analysis using the next generation sequencing (NGS) methodologies outlined below.

#### Whole exome sequencing

SGA samples underwent whole exome sequencing (WES) using the Agilent SureSelect Human All Exon V5 kit (Agilent Technologies Inc., Santa Clara, USA) by BGI (BGI Genomics, Hong Kong, China), and were sequenced with the high-throughput sequencing platform of Complete Genomics (Complete Genomics Inc., San Jose, USA) as detailed in Stalman et al. (44).

#### HaloPlex targeted NGS panel

A HaloPlex DNA targeted gene enrichment panel was designed using SureDesign software (Agilent Technologies Inc., Santa Clara, USA) to capture known and candidate genes involved in fetal growth restriction, including *SAMD9*. A detailed protocol has been described previously (45). In brief, FGR and POC samples were sequenced on a NextSeq sequencer (Illumina Inc., San Diego, USA) and analyzed with SureCall software (version 4.0.1.46, Agilent Technologies Inc., Santa Clara, USA) using the HaloPlex Default Method.

#### Nonacus targeted NGS panel

A Nonacus Cell3TM target NGS panel (Nonacus Ltd., Birmingham, UK) was designed to cover the coding regions of *SAMD9*. Nine samples from children with FGR and eight with SRS were sequenced on a MiSeq platform (Illumina Inc., San Diego, USA). Binary alignment map (BAM) files were produced by aligning FASTQ files to the GRCh38 reference genome with the Burrows-Wheeler Aligner (v0.7.17). Reads were grouped by unique molecular identifiers (UMIs) performed with fgbio (v0.4.0). Variant calling was performed with Platypus (v 0.8.1). Variant annotation was performed with Ensembl Variant Effect Predictor (VEP) and QIAGEN Clinical Insight (QCI). A detailed description has been previously reported (45, 46).

#### Prediction of pathogenicity

Individual variant pathogenicity was evaluated using SIFT (http://sift.jcvi.org/) and PolyPhen-2 (http://genetics.bwh.harvard.edu/pph2/), and CADD (Combined Annotation Dependent Depletion) score (https://cadd.gs.washington.edu).

#### Allele frequency

For each cohort, the *SAMD9* variant allele frequency was calculated by dividing the number of alleles with any specific variant by the total number of alleles sequenced. Allele frequencies were compared to data in gnomAD v2.1.1 (accessed May 2022, https://gnomad.broadinstitute.org) (47).

### Analysis of SAMD9 expression

RNA expression of *SAMD9* in a panel of adult human tissues was obtained from the Human Protein Atlas (https://www.proteinatlas.org) using the consensus RNA-Seq dataset raw counts (https://www.proteinatlas.org/ENSG00000205413-SAMD9/tissue) (48). Relative expression of *SAMD9* in a panel of human fetal and adult tissue was adapted from the previous report of Buonocore et al., 2017 (10). Single cell RNA sequencing analysis of placental *SAMD9* was evaluated using publicly available data at https://maternal-fetal-interface.cellgeni.sanger.ac.uk (49) and http://placenta.grid.wayne.edu/ (50).

### Graphical representations

Graphics were generated using GraphPad Prism version 8.4.3 for Windows (GraphPad Software, San Diego, USA; www.graphpad.com). Illustrations of SAMD9 protein domains were created using Domain Graph version 2.0 (51).

### Standard deviation score (SDS)

Birth SDS values, whenever not reported in the original article, were calculated using the PediTools Web calculator (https://peditools.org) (52).

### Statistical analysis

GraphPad Prism version 8.4.3 for Windows (GraphPad Software, San Diego, USA; www.graphpad.com) was used to perform Chi-squared tests for SAMD9 variants affecting arginine residues, and Mann-Whitney U tests for birth weight, gestational age and birth weight SDS data. Fisher’s exact test was used to test SAMD9 variant allele frequency in the studied cohorts compared to those reported in gnomAD v2.1.1.

## Results

### SAMD9 variants and endocrine features

**Figure 1:**
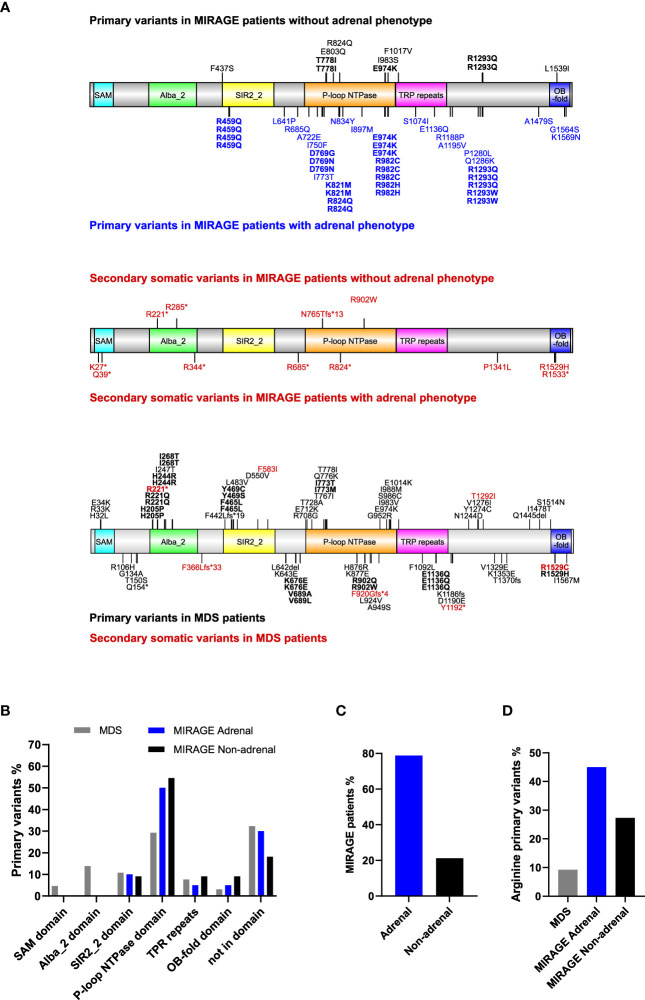
Overview of SAMD9 variants and relation to adrenal insufficiency. **(A)** Depiction of SAMD9 showing the variants reported in individuals with MIRAGE syndrome (top diagrams) and those with myelodysplastic syndrome (MDS) (bottom diagram). Predicted domains are indicated by different colors. Codons with multiple reported cases are shown in bold. Secondary somatic variants are represented in red. **(B)** Distribution of SAMD9 primary variants across the specific domains for individuals with MDS, and MIRAGE with or without adrenal insufficiency (Note: secondary somatic variants excluded). **(C)** MIRAGE patients reported to date with or without adrenal insufficiency. **(D)** Percentage of gain-of-function variants involving arginine residues in individuals with MDS and MIRAGE syndrome, with or without adrenal insufficiency.

Based on a review of the literature, a total of 116 individuals with likely pathogenic variants in *SAMD9* were identified. The distribution of all variants associated either with MIRAGE syndrome or MDS is shown in **Figure 1A**, with secondary somatic changes in SAMD9 indicated in red. Overall, 52 (44.8%) children had features of MIRAGE syndrome (including four individuals in two families), whereas 64 (55.2%) individuals presented with MDS (including five individuals in two families) (**Figure 1A**).

MIRAGE-associated variants tend to cluster within certain regions of the SAMD9 protein, especially the P-loop NTPase domain, whereas MDS-associated variants are distributed across the protein domain structure (**Figures 1A, B**). Certain “hotspot” variants and codons with recurrent changes were seen in all groups (**Figure 1A**). Although primary adrenal insufficiency has been considered one of the main features of MIRAGE syndrome since the identification of this multisystem disorder (41/52, 78.8%), a substantial number of children with MIRAGE features are now reported without an adrenal phenotype (11/52, 21.2%) (**Figure 1C**). Interestingly, changes in arginine residues are more prevalent in MIRAGE patients overall compared to MDS (21/51, 41.2%) MIRAGE; 6/65, 9.2% MDS, χ ^2^ = 16.33, P < 0.0001), and are potentially enriched in individuals with MIRAGE syndrome who have an adrenal phenotype, but numbers are small (18/40, 45.0% MIRAGE adrenal; 3/11, 27.3% MIRAGE non-adrenal, χ ^2^ = 1.119, P = 0.2901) (**Figure 1D**).

**Figure 2:**
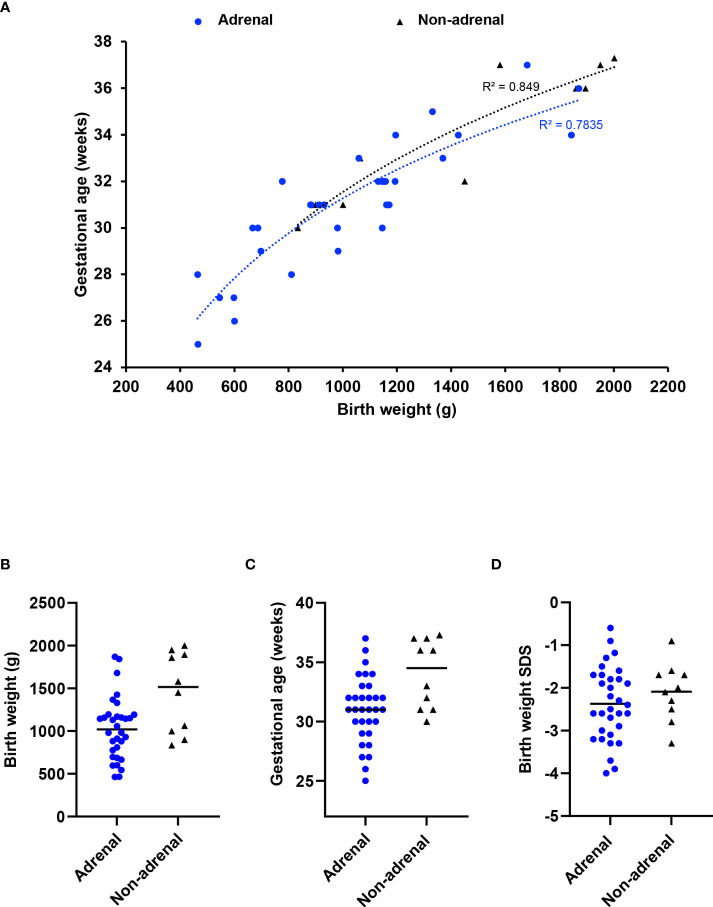
Relation between adrenal insufficiency and gestational age/birth weight. **(A)** Correlation plot between gestational age and birth weight at delivery for children with MIRAGE syndrome, with or without adrenal insufficiency. A logarithmic trendline and an R squared value for each phenotypic group is shown. Birth weight **(B)**, gestational age in weeks **(C)** and birth weight standard deviation score (SDS) **(D)** of children with MIRAGE syndrome reported to date, with and without adrenal insufficiency, with median values shown by the horizontal line.

To address whether children without an adrenal phenotype have a less severe condition in general, we used birth weight and gestational age as surrogate “markers” of phenotype severity. Infants with MIRAGE syndrome who do not have an adrenal phenotype tend to weigh more at birth (median weight 1515g, range 834 to 2002g) than those with adrenal features (median 1020g, range 464 to 1870g) (P < 0.05). They are delivered later (median age 34.5 weeks, range 30 to 37.3) compared to those having an adrenal phenotype (median 31 weeks, range 25 to 37 weeks) (P < 0.05) (**Figures 2A** **–C**). Birth weight SDS was not significantly different (adrenal phenotype, median -2.45, range -4.0 to -0.6; without adrenal phenotype, median -2.05; range -3.3 to -0.9; P = 0.34) (**Figure 2D**). Taken together, these data suggest that there may be a subtle spectrum of disease phenotypes, and that adrenal features are more common in very small, preterm babies with MIRAGE syndrome.

**Figure 3:**
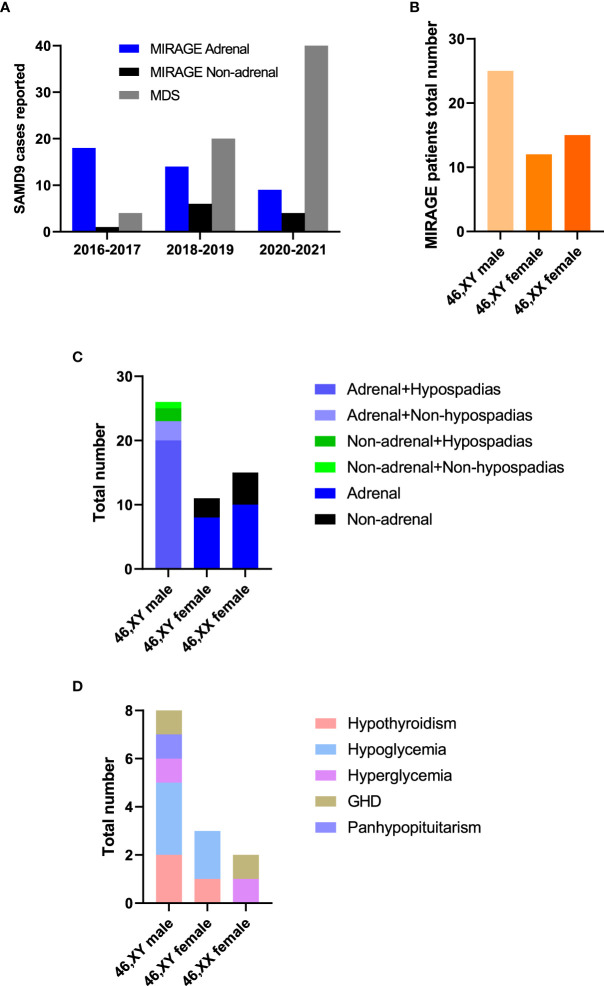
MIRAGE endocrine features. **(A)** Individuals with *SAMD9* pathogenic variants reported from 2016 to 2021. **(B)** Total numbers of MIRAGE patients based on karyotype and reported sex assignment. “46,XY female” indicates a 46,XY child who was brought up as a girl. **(C, D)** Endocrine features of children with MIRAGE syndrome, where reported. Data include two reported individuals with MIRAGE syndrome who developed endocrinopathies not originally described in the publication. GHD, growth hormone deficiency.

Since the first description of MIRAGE syndrome in 2016, there has been an increasing number of publications reporting patients with MIRAGE features with variants in *SAMD9*, and the phenotypic spectrum of SAMD9-related conditions has now expanded. Although adrenal insufficiency was a predominant feature in early reports, the relative proportion of children without adrenal insufficiency has increased with time (**Figure 3A**). Whilst 46,XY boys with hypospadias are now more common than 46,XY girls with severe gonadal dysfunction, 46,XX girls remain underreported (**Figures 3B, C**). Several patients have been identified who have additional endocrinopathies including hypothyroidism, hypo- and hyper-glycemia, growth hormone deficiency and panhypopituitarism, although it is often unclear how extensively most children have been investigated for endocrine disorders and some may have died before these were manifest (**Figure 3D**).

### Placental SAMD9 expression and recurrent miscarriage

**Figure 4:**
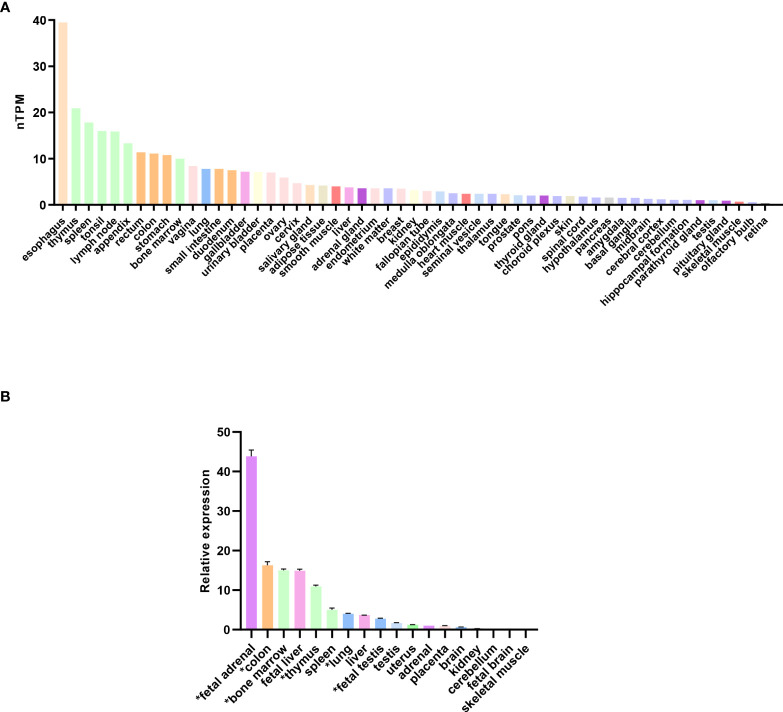
Expression of SAMD9 in human tissues. **(A)** RNA expression of SAMD9 in several adult human tissues. Data reproduced and modified with permission from the Human Protein Atlas (www.proteinatlas.org) (48). nTPM = normalized Transcripts Per Million. **(B)** SAMD9 expression in different human fetal and adult tissues showing high expression in fetal adrenal as well as in tissues affected in the clinical phenotype (adapted from Buonocore et al., 2017). Tissues especially relevant to the phenotype are highlighted (*). Data are shown as relative expression compared with GAPDH. Representative data are shown as mean ± SEM for a single study performed with triplicate technical replicates.

**Figure 5:**
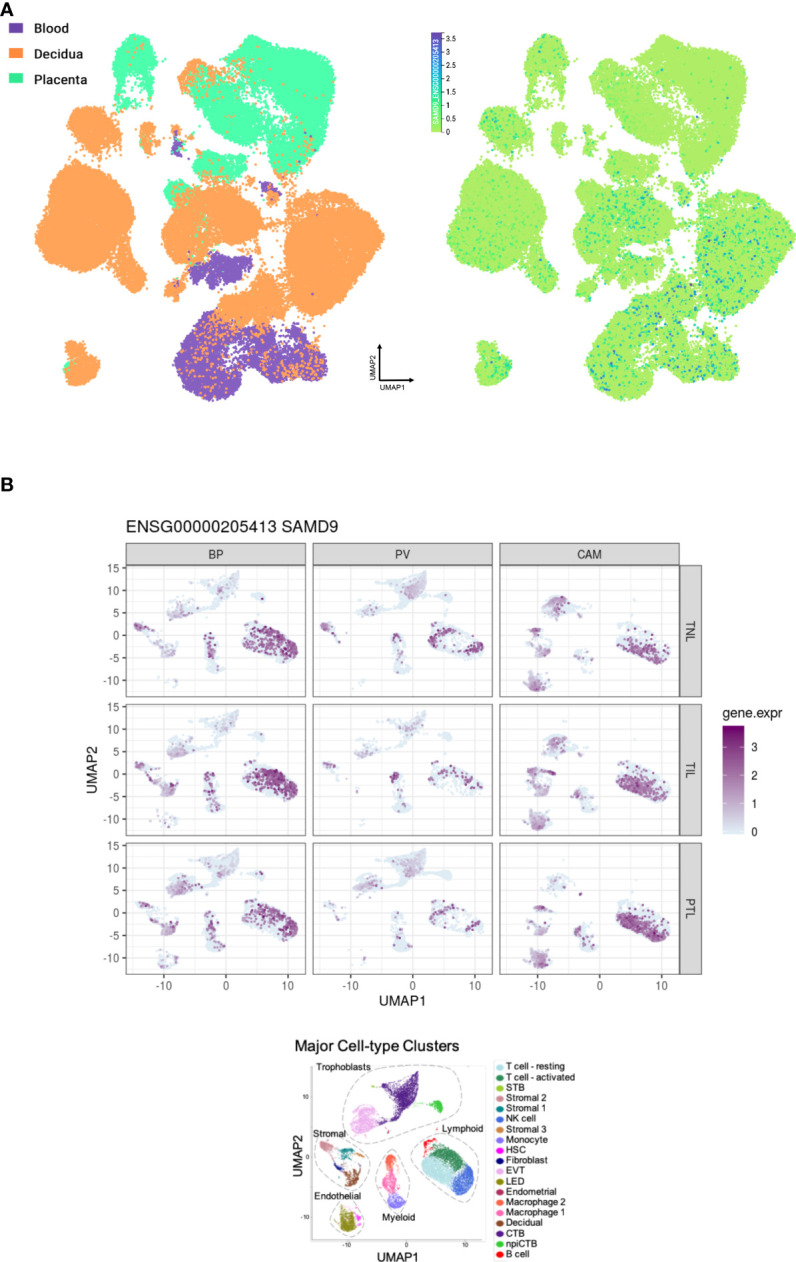
Expression of SAMD9 in human placenta. **(A)** Single cell RNA sequencing expression in first trimester placenta shown in the left panel in orange. Data accessed from https://maternal-fetal-interface.cellgeni.sanger.ac.uk/ (49). Higher color intensity is directly proportional to high levels of gene expression. **(B)** Expression of SAMD9 in single cell RNA data from late trimester human placenta. Highest expression of SAMD9 is in purple. Major cell-type clusters are indicated by different colors in the bottom panel. Reproduced from Pique-Regi et al., 2019. BP, Basal Plate; PV, Placental Villi; CAM, Chorioamniotic membranes; TNL, Term No Labor; TIL, Term In Labor; PTL, Preterm Labor; UMAP, Uniform manifold approximation and projection. Data accessed from http://placenta.grid.wayne.edu/ (50).

**Figure 6:**
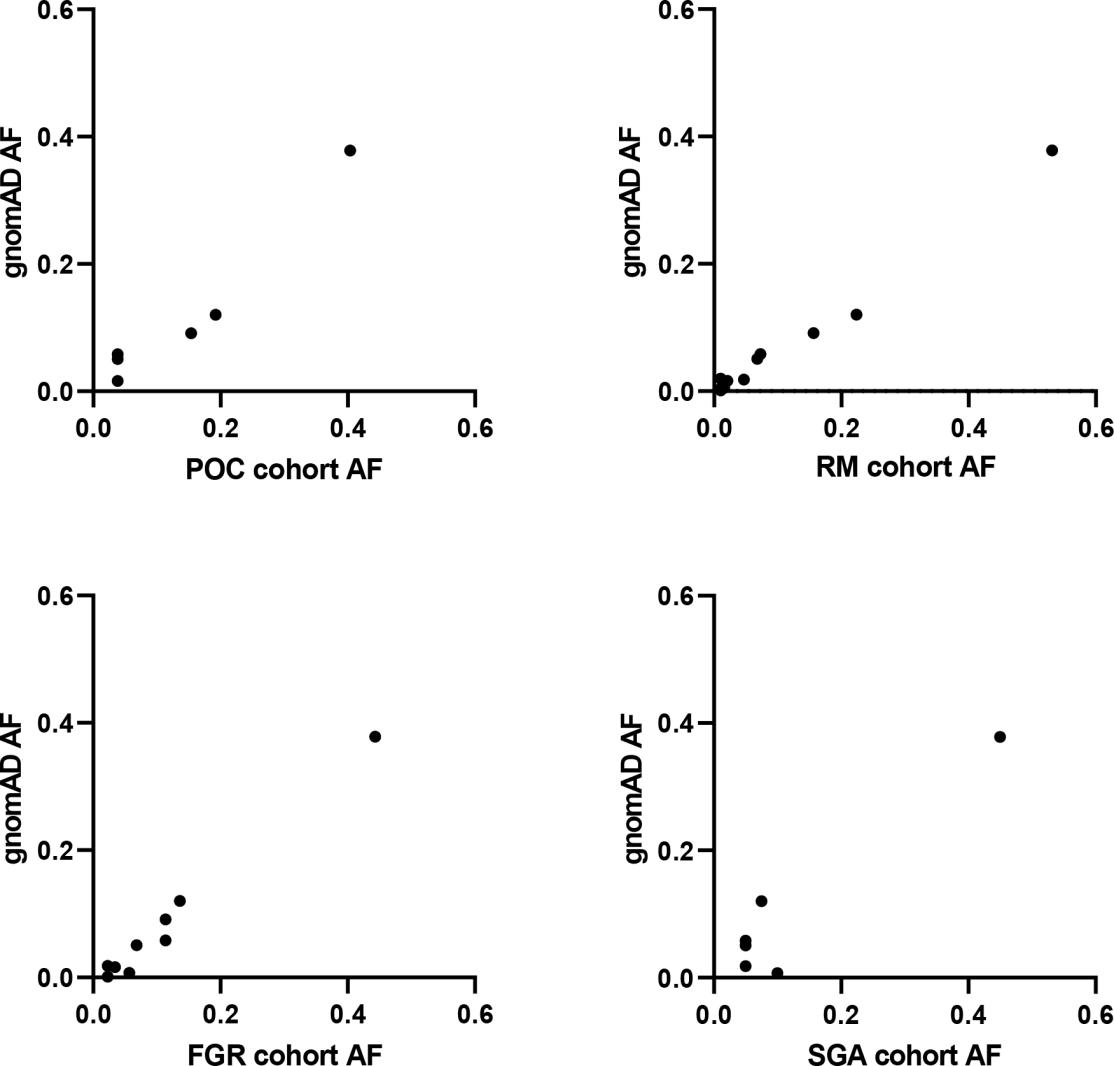
SAMD9 variants in studied cohorts. Correlation plots between allele frequency (AF) of *SAMD9* variants detected in our cohorts and that present in gnomAD. No data are shown for Silver-Russell Syndrome (SRS) as no variants in SAMD9 were detected. POC, Product of Conception; RM, Recurrent Miscarriage; FGR, Fetal Growth Restriction; SGA, Small for Gestational Age.

Analysis of RNA expression in several adult and fetal human tissues shows high levels of *SAMD9* in the esophagus, fetal adrenal, colon, bone marrow, thymus, lung and fetal testis (**Figures 4A, B**), which are tissues particularly affected in MIRAGE syndrome. FGR though, remains a common issue in MIRAGE syndrome and babies are often delivered due to severe FGR. *SAMD9* is a growth repressor and could be directly implicated in the pathogenesis of growth restriction in the fetus itself or might affect the fetoplacental unit. To address this further, we investigated the expression of *SAMD9* in human placenta using publicly available resources. Data from single cell RNA sequencing indicate that only a small population of annotated cells show *SAMD9* expression in first trimester human placenta (**Figure 5A**). In a study of third trimester placenta (33-40 weeks of gestation), more extensive *SAMD9* expression was seen especially in lymphoid tissues (**Figure 5B**). No enrichment of pathogenic nor polymorphic variants in *SAMD9* was identified in DNA from early lost pregnancies (POC), or in couples who had experienced recurrent miscarriage (RM) (**Table 2**; **Figure 6**).

**Table 2:** SAMD9 allele frequency in the study cohorts.

SAMD9 variants (p.)	VEP Annotation	CADD Score	Allele Frequency				
			gnomAD v2.1.1	POC (n=26)	RM (n=96)	FGR (n=44)	SGA (n=20)
**R75W**	missense	16.48	0.02	–	0.01	–	–
**S86F**	missense	<10	0.00	–	0.01	–	–
**I143T**	missense	<10	0.12	0.19	0.22	0.14	0.08
**F313**	synonymous	<10	–	–	0.01	–	–
**Y320**	synonymous	<10	0.38	0.40	0.53	0.44	0.45
**N449S**	missense	<10	0.02	0.04	0.05	0.02	0.05
**A454T**	missense	<10	0.05	0.04	0.07	0.07	0.05
**P466**	synonymous	<10	0.00	–	–	0.02	–
**T479M**	missense	<10	0.06	0.04	0.07	0.11	0.05
**V549L**	missense	21.80	0.09	0.15	0.16	0.11	–
**D881G**	missense	23.10	0.02	0.04	0.02	0.03	–
**K894E**	missense	<10	0.01	–	–	0.06	0.10
**N1003S**	missense	<10	0.00	–	0.01	–	–
**F1275**	synonymous	<10	0.02	–	0.02	–	–
**A1556T**	missense	10.12	0.01	–	0.02	–	–

Allele frequency for the variants detected in each cohort is shown. No data are shown for the Silver-Russell Syndrome (SRS) group as no variants were detected. Allele frequency is calculated by dividing the total number of alleles carrying the variant by the total number of alleles present in each individual cohort.

VEP, Variant Effect Predictor; CADD, Combined Annotation Dependent Depletion; FGR, Fetal Growth Restriction; SGA, Small for Gestational Age; POC, Products of Conception; RM, Recurrent Miscarriage.

### SAMD9 and growth restriction

Given the range of phenotypes associated with *SAMD9* that have now been reported, we investigated the potential role of *SAMD9* variants in a mixed cohort of children with FGR, SGA, and SRS (**Table 1**). Samples were subjected to targeted NGS and WES, with the aim of identifying any potential pathogenic variants and/or any enrichment of rare variants in *SAMD9* in any of the groups. We did not find any predicted pathogenic variants in the cohorts studied, and we did not observe any significant differences in *SAMD9* variant allele frequencies in our cohorts compared to those reported in gnomAD (Fisher’s exact test, all analyses P = 1) (**Table 2** and **Figure 6**).

## Discussion

MIRAGE defines a multisystem disorder caused by heterozygous missense variants in *SAMD9*, which is located on the long arm of chromosome 7. These variants lead to a gain-of-function of the growth repressor SAMD9, causing tissue hypoplasia and growth restriction. The natural history of the condition can be modified in the hematopoietic system at least by revertant somatic rescue mechanisms that can occur *in cis* to “remove” the affected allele. These events include somatic nonsense or frameshift variants, often only present in a small proportion of sequencing reads, and progressive loss of chromosome 7 or more specifically 7q, where *SAMD9* is located. Cells carrying the mutated *SAMD9*, which then develop the secondary somatic changes, have a clonal advantage allowing partial rescue. However, this can lead to the development of myelodysplasia in the hematopoietic system secondary to the loss of chromosome 7.

As is often the situation when new clinical associations are identified, the first MIRAGE patients reported exhibited the most severe phenotypic features. Many children died in the first few months of life or *in utero*. More recently additional children with MIRAGE have been described, many of whom have more variable or milder features, and an even larger cohort of children have been identified who present with MDS. Interestingly, MIRAGE phenotypic features have also recently been described in two patients carrying *SAMD9L* variants, which are associated with MDS and in some cases Ataxia pancytopenia (OMIM:159550) and CANDLE phenotypes (37). Here we show that children with MIRAGE features and growth restriction are more likely to have recurrent or “hotspot” variants compared to children with MDS, and more likely affecting amino acids in the P-loop NTPase domain or arginine residues, suggesting a potential emerging genotype-phenotype correlation. Of note, a SAMD9/9L effector domain (codons 134-385, which include the Alba domain) in association with DNA has recently been crystalized (53), but this structure does not contain most of the key hotspot codons relevant to MIRAGE syndrome.

Adrenal hypoplasia has been one of the key features of MIRAGE syndrome, with most children initially described having an adrenal phenotype. As more cases have been reported, it has emerged that an increasing number of patients do not have an adrenal phenotype, as we have shown here. In general, early adrenal dysfunction is associated with a lower birthweight and lower gestational age, as well as greater degree of gonadal dysfunction as indicated by the genital phenotype. Whether adrenal insufficiency contributes to prenatal fetal distress is unclear. Therefore, a spectrum of phenotypic severity seems to be emerging. This range of features could be linked to the underlying genetic variant; for example, on the limited data available, changes in codon 982 (p.R982C, p.R982H) seem to associate with adrenal dysfunction, whereas two children with changes in the neighboring codon (p.I983S) did not have adrenal features. More data are needed in this regard. Alternatively, some children without adrenal insufficiency could have adrenal rescue mechanisms such as somatic revertant mosaicism in progenitor cells that repopulate the gland under ACTH stimulation, but this theory is difficult to prove given the challenges in accessing adrenal tissue for investigation. On a practical level, adrenal features may be masked if a child receives steroids for another reason (e.g., chronic lung disease or infection), although to our knowledge this was not the situation in the children described here. Finally, whether any of these individuals develop adrenal insufficiency with time is unknown, so clinical vigilance and potentially periodic screening of adrenal function may be warranted.

The association between growth restriction and primary adrenal insufficiency is not limited to MIRAGE syndrome. Similar growth restriction syndromes characterized by adrenal insufficiency, FGR and other features, include IMAGe syndrome (intrauterine growth restriction, metaphyseal dysplasia, adrenal hypoplasia, genitourinary anomalies), caused by gain-of-function variants in the cell-cycle repressor *CDKN1C* (54, 55), and “IMAGe-like” syndrome with immunodeficiency, caused by biallelic variants in the DNA polymerase *POLE1* (56). To date, current UK data suggest that *SAMD9*-related conditions have not been found in children with primary adrenal insufficiency of unknown etiology without prematurity or growth restriction (57).

In addition to the well-described gonadal defects that can affect both the testis and ovary (15), a range of additional endocrine features have been reported with MIRAGE syndrome and SAMD9 disruption. These include glucose dysregulation, hypothyroidism, and hypothalamo-pituitary issues such as growth hormone insufficiency and panhypopituitarism. Sometimes these features may be influenced by the clinical status of the child (especially in pre-term babies) or by hypocortisolemia. However, it does seem that additional endocrine features can occur, and these may well be underreported if they are not regularly assessed or if children do not survive long enough for them to manifest. More long-term data are needed in this regard, including whether puberty is affected in MIRAGE syndrome, and how best to optimize growth potential throughout childhood and adolescence.

As prenatal and postnatal growth issues are common in MIRAGE patients, and infants are often delivered early due to severe FGR, we investigated a possible link between SAMD9 and pregnancy loss. Data from single cell RNA sequencing show low levels of SAMD9 expression in first trimester human placenta, suggesting that it is unlikely to be a driver of growth restriction *via* a placental mechanism during that time, but rather *via* a fetal specific system. However, higher expression was seen later in pregnancy, and histological evaluation of placenta tissues from SAMD9 patients showed poorly developed distal villous trees with widening of the intervillous space, suggesting abnormal development (22). These findings could indicate a role of SAMD9 in both the placenta and fetus, having therefore a dual function in prenatal growth disruption. In our analysis of a limited number of available samples, we did not identify likely pathogenic *SAMD9* variants in early POC tissue (spontaneous pregnancy loss), nor in genomic DNA of couples who had early recurrent miscarriages (RM). Mosaic placental mechanisms occurring *de novo* in the fetus could not be excluded. This contrasts with *CDKN1C*, which shows marked expression in first trimester placental tissue, highlighting a potential role for fetal growth and survival (45). Taken together, the higher placental expression of *SAMD9* in later pregnancy coupled with evidence of abnormal placental development in affected patients, could be contributing factors to poor growth and fetal distress seen in MIRAGE syndrome, and a reason these babies are delivered early.

*SAMD9* variants can be found in children born SGA (22) and interestingly, they can be inherited from asymptomatic parents, exhibiting different rescue mechanisms from offspring (20). This led us to analyze *SAMD9* in samples from a cohort of children with FGR, SGA and SRS, to investigate whether potential predicted pathogenic variants could be found that could explain the growth restriction phenotype. We did not find any primary or secondary somatic variants, nor did we observe an enrichment of more common variants in *SAMD9* in the cohort studied, suggesting that *SAMD9* is not a frequent driver of growth disorders unless other features are present.

This study has several important implications. Establishing the range of endocrinopathies in MIRAGE syndrome is essential to reach a quick diagnosis and tailor patient treatment, as adrenal insufficiency is a life-threatening condition and thyroid hormone imbalance can affect brain development and growth. It is also important to define and treat other features, such as dry eyes, feeding difficulties and esophageal reflux, periodic fever, immune dysfunction and gastrointestinal effects, amongst other considerations (11). Establishing a diagnosis of a SAMD9-related condition enables monitoring of monosomy 7, as a secondary somatic event to remove the primary driver, and consequent potential MDS risk. Finally, the features of MIRAGE syndrome can be very non-specific and often seen in sick babies in neonatal medicine delivered prematurely with growth restriction. Raising the awareness of this condition and having greater access to rapid genetic sequencing could be essential to identify children who harbor pathogenic *SAMD9* variants, and to identify some of the “missing” cohort of 46,XX girls with this condition. Indeed, more widespread use of whole exome/genome sequencing for children with growth restriction and associated features is likely to identify more children with MIRAGE-associated conditions and provide more information on the range of phenotypic features.

This study has several limitations: 1) Following the literature review, several case reports lacked complete information such as BW and GA, especially for patients carrying *SAMD9* variants in the MDS cohort. Furthermore, detailed genetic studies of genotype-phenotype co-segregation were not always described in MDS patients, and functional work is limited suggesting that some false positives may have been reported. 2) As discussed above, endocrine features may also be overlooked and not reported, or they may develop later with time. There is therefore a need for a life-course data analysis to be able to capture the full range of phenotypes associated with *SAMD9* variants. 3) Due to a relatively small cohort of miscarriage tissues and RM couples, we might have missed *de novo* variants, but potentially parental germline mosaicism would be enriched, and this is important as there are several sibling pairs with *SAMD9* variants reported. 4) The FGR cohort analyzed in this study was relatively small. Analysis therefore does not exclude SAMD9 as a potential pathogenic gene in this cohort, but suggests it is not a common occurrence.

SAMD9-associated variants comprise a wider spectrum of phenotypes than originally reported, including children with neonatal growth restriction and multisystem features but without adrenal insufficiency. Endocrinopathies are also emerging, though these are possibly overlooked. Indeed, as these patients may present to a diverse range of health care professionals, and with features beyond those in the classic MIRAGE acronym, perhaps referring to “SAMD9-related syndromes” in the future may be more appropriate. We have also shown that *SAMD9* variants are not common in fetal growth disorders unless additional features are present. Nevertheless, monitoring severe FGR children remains essential and has important implications in reaching a specific diagnosis for personalized multidisciplinary management.

## Data availability statement

The original contributions presented in the study are publicly available and can be found here http://doi.org/10.17605/OSF.IO/WMVXY (39). These data include an overview of SAMD9 publications and linked reported variants, and a summary of all SAMD9 variants identified using the HaloPlex, Nonacus and WES platforms.

## Ethics statement

The studies involving human participants were reviewed and approved by BBB Research Ethics Committee references: 09/H0405/30 and 09/H0405/30+5; Moore cohort reference: 2001/6029. Written informed consent to participate in this study was provided by the participants’ legal guardian/next of kin.

## Author contributions

JS and FB designed the targeted NGS panels. JS carried out NGS experiments. MI and ID conducted bioinformatic pipeline analysis. SS provided variants for the SGA samples. NS coordinated samples and clinical data from the BBB. EW contributed to the clinical evaluation of the article. GM is the founder of the BBB and Moore Cohort and provided all the samples and conceptual input. JA and FB undertook literature reviews, conceptualized, and wrote the article. FB conducted the molecular analysis, produced figures and tables. All authors contributed to manuscript revision, read, and approved the submitted version.

## Funding

This research was funded in whole, or in part, by the Wellcome Trust (grant 209328/Z/17/Z). For the purpose of Open Access, the author has applied a CC BY public copyright license to any Author Accepted Manuscript version arising from this submission. JA also has research support from Great Ormond Street Hospital Children’s Charity (grant V2518) and the National Institute for Health Research, Great Ormond Street Hospital Biomedical Research Centre (grant IS-BRC-1215-20012). The views expressed are those of the authors and not necessarily those of the National Health Service, National Institute for Health Research, or Department of Health.

## Acknowledgments

We would like to thank the Wellbeing of Women Baby Bio Bank (BBB) (University College London, Imperial College London Wellbeing of Women) for providing the POC, RM, FGR, SGA and SRS samples.

## Conflict of interest

The authors declare that the research was conducted in the absence of any commercial or financial relationships that could be construed as a potential conflict of interest.

## Publisher’s note

All claims expressed in this article are solely those of the authors and do not necessarily represent those of their affiliated organizations, or those of the publisher, the editors and the reviewers. Any product that may be evaluated in this article, or claim that may be made by its manufacturer, is not guaranteed or endorsed by the publisher.
